# Small volatile lipophilic molecules induced belowground by aphid attack elicit a defensive response in neighbouring un-infested plants

**DOI:** 10.3389/fpls.2023.1154587

**Published:** 2023-06-23

**Authors:** Pasquale Cascone, Jozsef Vuts, Michael A. Birkett, Sergio Rasmann, John A. Pickett, Emilio Guerrieri

**Affiliations:** ^1^ Institute for Sustainable Plant Protection, Consiglio Nazionale delle Ricerche, Portici, Napoli, Italy; ^2^ Biointeractions and Crop Protection Department, Rothamsted Research, Harpenden, Hertfordshire, United Kingdom; ^3^ Institute of Biology, University of Neuchatel, Neuchatel, Switzerland; ^4^ School of Chemistry, Cardiff University, Cardiff, United Kingdom; ^5^ Institute for Sustainable Plant Protection, Consiglio Nazionale delle Ricerche, Torino, Italy

**Keywords:** root exudates, plant-plant communication, indirect defence, sulcatol, sulcatone, aphid parasitoid, 1-octen-3-ol

## Abstract

In pioneering studies on plant-aphid interactions, we have observed that *Vicia faba* plants infested by aphids can transmit signals via the rhizosphere that induce aboveground defence in intact, neighbouring plants. The aphid parasitoid *Aphidius ervi* is significantly attracted towards intact broad bean plants grown in a hydroponic solution previously harbouring *Acyrtosiphon pisum*-infested plants. To identify the rhizosphere signal(s) possibly mediating this belowground plant-plant communication, root exudates were collected using Solid-Phase Extraction (SPE) from 10-day old *A. pisum*-infested and un-infested *Vicia faba* plants hydroponically grown. To verify the ability of these root exudates to trigger defence mechanisms against the aphids we added them to *V. fabae* plants grown in hydroponic solution, and tested these plants in the wind-tunnel bioassay to assess their attractiveness towards the aphids’ parasitoids *A. ervi.* We identified three small volatile lipophilic molecules as plant defence elicitors: 1-octen-3-ol, sulcatone and sulcatol, in SPE extracts of *A. pisum*-infested broad bean plants. In wind tunnel assays, we recorded a significant increase in the attractiveness towards *A. ervi* of *V. faba* plants grown in hydroponic solution treated with these compounds, compared to plants grown in hydroponic treated with ethanol (control). Both 1-octen-3-ol and sulcatol have asymmetrically substituted carbon atoms at positions 3 and 2, respectively. Hence, we tested both their enantiomers alone or in mixture. We highlighted a synergistic effect on the level of attractiveness towards the parasitoid when testing the three compounds together in respect to the response recorded against them singly tested. These behavioural responses were supported by the characterization of headspace volatiles released by tested plants. These results shed new light on the mechanisms underlying plant-plant communication belowground and prompt the use of bio-derived semiochemicals for a sustainable protection of agricultural crops.

## 1 Introduction

For a long time, public and private organizations, the scientific community and society as a whole have called for sustainable agricultural practices that ensure food security for the growing human population while protecting biodiversity and the environment (UE, 2009). However, most pest management programs still rely consistently on the repeated application of synthetic pesticides. Without finding suitable alternatives, pest control will result in increased pesticide use with all associated drawbacks on human health and on the environment.

Plants communicate with other living organisms via chemical signals, which can be produced constitutively, or can be induced in response to abiotic and biotic stresses. These signals travel through the air and the soil and regulate the interactions between plants and all other organisms (Bruin and Dicke, 2001; Karban et al., 2014; Erb et al., 2015). The rhizosphere, which is the area of soil in close proximity to plant roots, is a site of significant chemical signalling, as roots release substances such as enzymes, mucilage, and various metabolites into the soil (Rovira, 1969). These substances, known as root exudates (Bais et al., 2003; Walker et al., 2003; Biedrzycki et al., 2010), contain both high molecular weight compounds, such as polysaccharides and proteins, and low molecular weight compounds, which can be further classified as primary metabolites (*e.g*. sugars, amino acids, and organic acids) and secondary metabolites (*e.g.* terpenes, flavonoids, glucosinolates and alkaloids) (Bais et al., 2003; Canarini et al., 2019; Ehlers et al., 2020; Chai and Schachtman, 2022).

Belowground, these compounds play critical roles in various biological processes (Badri and Vivanco, 2009; Weir et al., 2010), and regulate the interactions between plants and microbes (Weir et al., 2010), plants and animals (Johnson and Rasmann, 2015) and between plants (Bais et al., 2006). Research has also provided evidence that root exudates are a sort of “wireless” signals in cross-plant communication (Sharifi and Ryu, 2021), as they persist in the soil for extended periods and affect neighbouring plants as well as future generations by altering soil properties (referred to as plant-soil feedback). In addition to water-soluble molecules, roots also release lipophilic volatile organic compounds (VOCs) that diffuse through the gaseous medium, and also interact with substrate particles and moisture in the substrate pore spaces (Som et al., 2017). There is evidence that belowground plant-plant communication can be used to warn the neighbouring conspecifics about the presence of an insect herbivore, and to give them the opportunity to respond promptly and effectively to the pests (Cascone et al., 2023). This type of communication is regulated by the release of elicitor/s by the roots of infested plants that are adsorbed and systemically translocated by neighbouring conspecifics. It is hypothesized that plants produce unique blends of molecules in response to insect herbivory that could elicit specific responses in neighbours whilst travelling through the soil matrix (Baetz and Martinoia, 2014; Haichar et al., 2014).

VOCs emitted by plants, primarily studied as aboveground chemical signals, are receiving increasing attention. In fact, a growing body of research has recently shown that VOCs also play an important role in belowground plant-plant interactions (Rasmann and Turlings, 2016; Gfeller et al., 2019; Rasmann and Hiltpold, 2022). Root-emitted VOCs have been shown to impact the belowground microbial community (Weisskopf et al., 2016), soil nematodes (Rasmann et al., 2005) as well as the behaviour of herbivorous insects (Robert et al., 2012).

In some systems, positive intraspecific (Chamberlain et al., 2001; Guerrieri et al., 2002) and interspecific (Li et al., 2016; Liu et al., 2017; Li et al., 2021) plant-plant belowground interactions have been demonstrated. For instance, it has been shown that un-infested broad bean (*Vicia faba*) plants that are grown in close proximity to aphid-infested plants become more attractive to aphid parasitoids than plants grown with healthy plants (Guerrieri et al., 2002). This increased attractiveness was not observed when the root contact was prevented, suggesting that the communication occurred belowground (Guerrieri et al., 2002). These findings were confirmed using hydroponic growing conditions, where un-infested *V. faba* plants placed in a hydroponic solution previously used to grow aphid-infested plants became attractive to parasitoids, while those placed in a hydroponic solution previously used to grow healthy plants did not change in attractiveness (Guerrieri et al., 2002). Similarly, an interspecific positive interaction has been studied highlighting how maize root exudates enhance broad bean nodulation and nitrogen fixation (Li et al., 2016; Liu et al., 2017; Li et al., 2021).

Although several lines of evidence suggest that *V. faba* plants are able to communicate through radical exudates, to the best of our knowledge, only one compound has been discovered to elicit a defence mechanism in neighbouring plants (Cascone et al., 2023). In a series of plant-plant communication experiments, it was found that the non-protein amino acid l-DOPA was one of the hydrophilic elicitors released by the roots of aphid-damaged *V. faba* plants. When treated with l-DOPA, healthy plants had altered aboveground VOC profiles and attracted more aphid parasitoids than untreated plants. This research emphasized the discovery of hydrophilic root signals that plants use to communicate with each other, but it may also be important to pay attention to Small Volatile Lipophilic Molecules (SVMLs) that are frequently found amongst the secondary metabolites released by plants (Birkett and Pickett, 2014). Hence, we here studied the possible role of SVMLs released by aphid-infested broad bean plants in plant-plant communication belowground that could be exploited for the sustainable protection of agricultural crops.

Aphids (Hemiptera: Aphidoidea), the most abundant invertebrate phloem-feeders, cause significant economic damage either by directly consuming plant sap or indirectly by transmitting phytopathogenic viruses (van Emden et al., 2007). Currently, the fight against these pests mainly relies on insecticides which still represent the most effective and cheapest method to fight them. But today, it has become essential to identify ecologically sustainable alternatives, with lower impact on the environment. In this regard, chemicals that are found to induce defence responses in intact plants can be used as new plant activators to trigger defence mechanisms when pest control is needed. For instance, SVMLs affecting the behaviour of insect parasitoids can be profitably exploited for the sustainable protection of crops by attracting the natural antagonists of insect pests (Pickett et al., 2013).

Here, we have identified three SVMLs released by *V. faba* roots upon aphid attack: 1-octen-3-ol, sulcatone and sulcatol. Both 1-octen-3-ol and sulcatol. Both 1-Octen-3-ol and Sulcatol have asymmetrically substituted carbon atoms at positions 3 and 2, respectively. Therefore, the enantiomers of each compound were tested individually and in racemic mixtures to assess their effect on plant attractiveness towards the aphid parasitoid *Aphidius ervi* (Hymenoptera: Braconidae). Finally, the most active compounds were tested in combination with each other to investigate any potential synergistic effect. The VOCs released aboveground by treated and control plants, potentially serving as attractant for the parasitoids, were also characterized.

## 2 Materials and methods

### 2.1 Insects

The parasitoid *A. ervi* was reared on its natural host, the pea aphid *Acyrthosiphon pisum* maintained on potted broad bean (*V. faba*) plants, cv. Aquadulce (Guerrieri et al., 1993). Aphid and parasitoid cultures were kept in separate environmental chambers at 20 ± 1°C, 75 ± 5% relative humidity and 18L: 6D photoperiod. Insect parasitoids used in the bioassays were reared as synchronized cohorts by exposing heavily infested plants for 24hr to 1-day old mated females; after a week, the resultant mummies were clipped from the plant and isolated in glass test tubes (60 × 8 mm) plugged with cotton wool. Experimental females were used within the first day after emergence, mated, and fed with a 50% honey solution. All experiments were conducted 3 hr from the onset of the photophase.

### 2.2 Plants

All bioassays were run using plants grown in hydroponic solution. *V. faba* seeds were soaked in water for 24 h, then potted in vermiculite and kept in a controlled environment room at 20°C. After 5 days, the seedlings were gently removed from the vermiculite, the seed coat discarded and the roots rinsed with water, carefully removing any vermiculite residue. Two seedlings were then placed in a glass beaker containing hydroponic solution made with Murashige and Skoog basal salt mixture (2 g L^-1^, Duchefa Biochemies, The Netherlands) and placed in a glasshouse (20°C, L:D 16:8 hr). Each beaker was wrapped in aluminium foil to hold the plants in position and to prevent the light from reaching the roots. The hydroponic solution was renewed every 2-3 days.

### 2.3 SVLMs chemical identification

To isolate potential SVLMs elicitors from roots, plants were grown as above. At the age of 11-12 days, at least 100 A*. pisum* of mixed age were placed onto each plant couple for the infested plants, and infested and non-infested plants were kept in separate rooms under the same climatic conditions (n=8). After 3 days, the hydroponic solution from the beakers was pooled separately for infested and non-infested plants in large bottles, which were connected through PTFE (polytetrafluoroethylene) tubing to 6 mL glass SPE cartridges containing C2/ENV+ sorbent used for trapping small polar and lipophilic compounds from water (Biotage, 941-0070-L). Cartridges were attached to vacuum pumps and conditioned by passing 2 column length of each of HPLC methanol then HPLC water through. PTFE tubing was washed with HPLC water. Hydroponic solution was added to the cartridges, which were connected to the holding bottles through PTFE tubing. After all the hydroponic solution has been passed through the cartridge columns by vacuum, each of them was flushed with HPLC water then left to run dry for 2 min. A column length of redistilled ether was pulled through each cartridge by vacuum and collected in vials. Ether extracts were dried with magnesium sulphate, filtered and concentrated to 500 mL under a stream of nitrogen. Samples were stored at -20°C until analysis. Extracts were analysed on an Agilent 6890A gas chromatograph (GC) equipped with a cool on-column injector, a flame ionization detector and a 30 m × 0.32 mm ID, 0.5 μm film thickness DB-WAX column (Agilent, Santa Clara, CA, USA). The oven temperature was maintained at 30°C for 1 min, then programmed at 5°C/min to 150°C and held for 0.1 min, then programmed at 10°C/min to 250°C and held for 20 min. Quantification of compounds was achieved by the single-point external standard method with a series of C7-C22 alkanes, where the amount of an analyte was estimated using the peak area of the nearest alkane peak, the amount of which was known. GC-MS analysis was done on a Micromass Autospec Ultima magnetic sector mass spectrometer (Waters, Milford, MA). The GC (Agilent 6890 N) was fitted with a 30 m × 0.32 mm i.d. × 0.5 μm film thickness DB-WAX column (Agilent, Santa Clara, CA, USA). Ionization was by electron impact (70 eV, 220°C). The GC oven temperature was maintained at 30°C for 5 min and then programmed to increase at 5°C/min to 250°C, with a 70-min run time. The identity of peaks was confirmed by comparison of their GC and MS properties with those of authentic standards and by GC peak enhancement using authentic samples [Sigma-Aldrich UK: sulcatone >99%, (S)-1-octen-3-ol >95%, BOC Sciences: (R)-1-octen-3-ol 98%, (R)-sulcatol >90%, Alfa Chemistry: (S)-sulcatol 96%.

### 2.4 Wind tunnel bioassays on plants treated with SVLMs

For assessing the effect of SVLMs on the indirect defence, 20 μl of each SVLM: (a) *R*-sulcatol, (b) S-sulcatol, (c) sulcatone, (d) *R*-1-octen-3-oI, (e) S-1-octen-3-ol, their racemic mixtures (a+b, d+e) and their combination a+c+d were added to beakers with 200 μl of clean hydroponic solution. Two seedlings were transferred into it and kept as described above for 24hr before testing them in the wind-tunnel. Plants treated with 20 μl of Ethanol were used as relative control. For each elicitor and their combinations, a total of ten plants grown in hydroponic solution were used and tested in a wind-tunnel (see Guerrieri et al., 1999 for details) daily in a random order to reduce any bias related to the time of the experiments. One hundred Parasitoid females were tested singly for each target in no-choice experiments, and observed for a maximum of 5 min. The percentage of response (oriented flights, landings on the target) to each target plant was calculated. The parameters of the bioassay were set as follows: temperature, 20 ± 1°C; 65 ± 5% RH; wind speed, 25 ± 5 cm s-1; distance between releasing vial and target with 50 cm at releasing point, ligh: 700 µmol m^2^ s^-1^.

### 2.5 Air entrainment of plants treated with SVLM

After broad bean plants were grown in hydroponic solution for 10 days, the hydroponic solution was replaced and treated with SVLM solutions or their combinations (20 µL) or ethanol (control, 20 µL) (n=4 replicates/treatment). After 24 h, the bean plants were enclosed in Multi-Purpose Cooking Bags [poly(ethyleneterephthalate)] or PET, volume 3.2 L, ~12.5 µm thickness, max. 200°C, Sainsbury’s Supermarkets Ltd., London, UK]. The bottom of the bag was enclosed around the top of the beaker containing the hydroponic solution. The inlet was fitted to the open end of the bag, and the outlet was fitted to a corner of the bag after cutting off with scissors. Air that had been purified by passage through an activated charcoal filter (BDH, 10-14 mesh, 50 g) was pushed into (750 mL/min) and pulled (700 mL/min) out of the bags. Volatiles were trapped onto Tenax (50 mg; Supelco, Bellefonte, USA) held in glass tubing (5 mm outer diameter) by two plugs of silanised glass wool. The Tenax tubes were conditioned by washing with dichloromethane (2 mL), followed by redistilled diethyl ether (2 mL) and heating at 132°C for 2 h under a stream of purified nitrogen. After 24 h sampling, the Tenax tubes were sealed in glass ampoules in an atmosphere of nitrogen and stored at -20°C until analysis. For VOC sample analysis, the Tenax tubes were inserted into the OPTIC PTV unit of a GC (30->250°C ballistically at a rate of 16°C/s) connected to a Micromass Autospec Ultima magnetic sector mass spectrometer (Waters, Milford, MA). The GC (Agilent 6890 N) was fitted with a 50 m × 0.32 mm i.d. × 0.52 μm film thickness HP-1 column (Agilent, Santa Clara, CA, USA). Ionization was done by electron impact (70 eV, 220°C). The GC oven temperature was maintained at 30°C for 5 min and then programmed to increase at 5°C/min to 250°C, with a 70 min run time. The identity of peaks was confirmed by comparison of their GC and GC–MS properties with those of authentic standards (see Sasso et al., 2007 for details), and by GC peak enhancement using authentic samples.

### 2.6 Statistical analyses

We used R4.2.2 (R Core Team, 2022) for all statistical analyses. To investigate whether aphid infestation can influence the emission of SVLMs by root plant we have analysed their amount by ANOVA followed by *post-hoc* Tukey’s multiple comparison test. To investigate whether treatments can affect the emission VOCs, we analysed the amount of each VOC using a Dirichlet regression and the R package “DirichletReg” (Maier, 2014). We analysed the proportion of each VOC peak area in relation to total peak areas as the dependent variable and treatments as independent variable. A Dirichlet regression is a type of compositional analysis based on the Dirichlet distribution and does not assume a multivariate normal distribution or homoscedasticity of the data (Douma and Weedon, 2019). Dirichlet regression, being a compositional analysis, uses a logarithmic link function of the compositional variable. This transformation overcomes potential problems with non-independence of proportional data (Douma and Weedon, 2019). Because of this transformation, it is not possible to have zeros in the data, therefore we replaced them by a small value using the DR_data function (Maier, 2014). The volatile emission patterns, measured as peak areas, were analysed through multivariate data analysis using PSL-DA (Projection to Latent Structures Discriminant Analysis) using the R package “ropls” (Thévenot et al., 2015). To investigate whether treatments can influence the behavioural response of the *A. ervi* females towards broad bean plant in the Wind-Tunnel assay, we used the R pa”kage " Agr”colae” (de Mendiburu, 2021). To determine if the proportions between: females make oriented flight towards plant vs un-oriented, and females landed on plant vs total vary with treatments, we used the Chi-square test followed by Ryan’s multiple range test (Fukushima et al., 2021).

## 3 Results

### 3.1 Sulcatone, sulcatol and 1-octen-3-ol are released as SVLM after aphid infestation in *Vicia faba*

A preliminary ether extraction of *V. faba* hydroponic solution showed Sulcatone (KI 1346), 1-octen-3-ol (KI 1449) and sulcatol (KI 1465) peaks were increased by aphid infestation (**Supplementary Figure S1**). This assay was replicated 9 times highlighting that the above-cited SVLMs were produced in higher significant amounts in infested *V. faba* plant compared to the un-infested ones (**Table 1**). Sulcatone is released 6-times more by root of infested *V. faba* plants compared to un-infested (Anova, df =1, F=120.007, P<0.001). Similarly, 1-octen-3-ol (Anova, df=1, f=12.3208, P=0.004) and sulcatol (Anova, df=1, f= 89.571, p<0.001) are released 9-times and 11-times more by infested *V. faba* roots, respectively (**Table 1**).

**Table 1:** SVLMs identified.

SVLM	Uninfested (n=8)	Infested by aphids (n=8)
	(µg ± SE)	
**sulcatone**	0.033 ± 0.003^a^	0.189 ± 0.013^b^
**1-octen-3-ol**	0.025 ± 0.045^a^	0.220 ± 0.034^b^
**sulcatol**	0.046 ± 0.031^a^	0.519 ± 0.038^b^

Mean of compound (µg ± SE) released by *Vicia fabae* roots in hydroponic culture. Letters were assigned by ANOVA and Tukey’s multiple comparison test (P ≤ 0.05).

### 3.2 Sulcatone, sulcatol and 1-octen-3-ol induce indirect defence in *Vicia faba*

**Figure 1:**
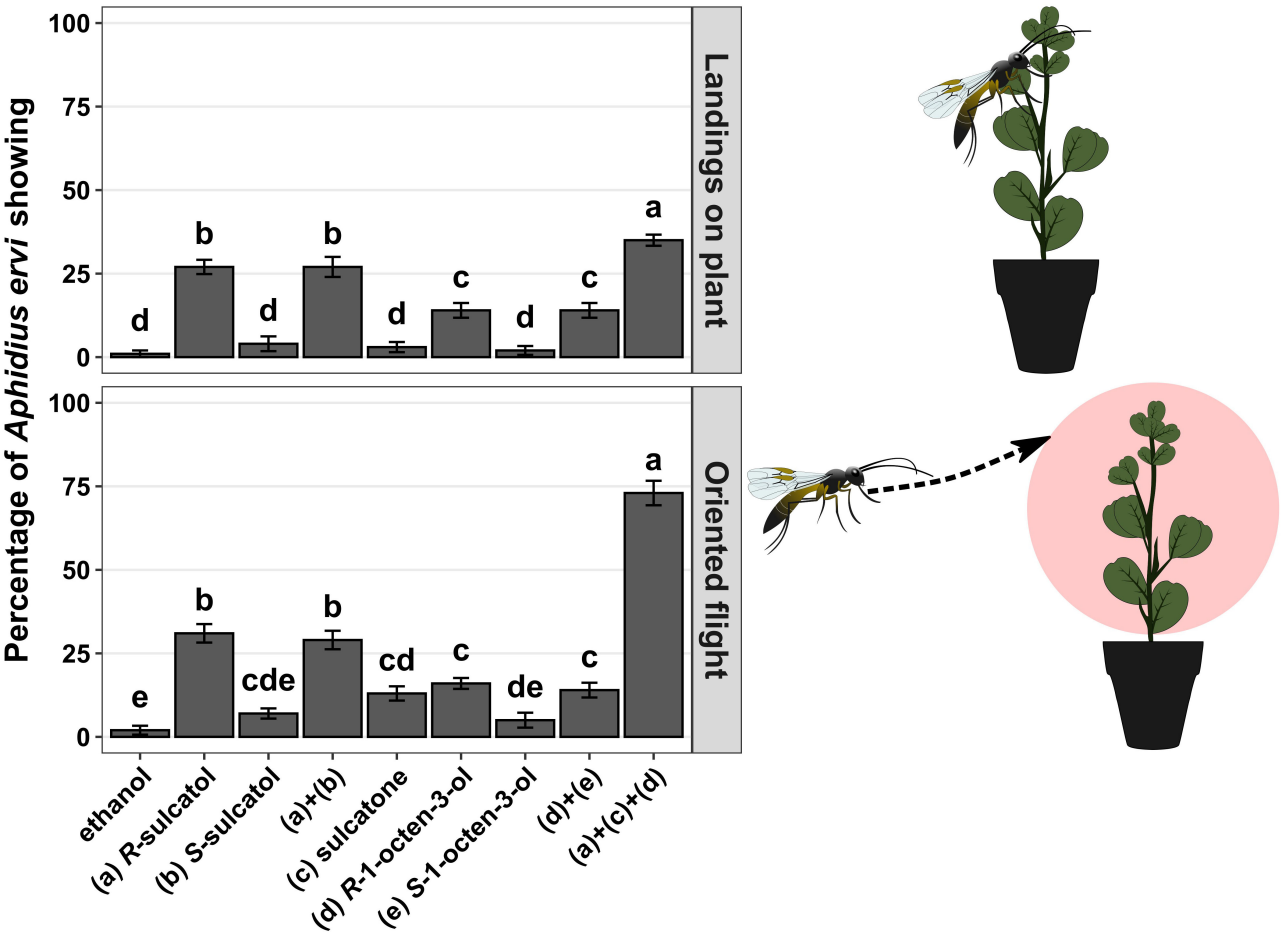
Wind tunnel bioassays. Different treatments with individual SVLMs (Small Volatiles Lipophilic Molecules) and their combinations in hydroponic culture of *Vicia fabae* are reported on x-axis. Percentage of *Aphidius ervi* females showing oriented flight and landing on source (plant) are reported on the y-axis. Different letters above bars indicate a significant difference between values (Ryan’s multiple range test for proportions after χ^2^ test, p < 0.05) (n = 10 for each).

Using wind-tunnel bioassays, we show that all identified root SVLMs and their combinations can induce an indirect defence response (**Figure 1**) in treated plants in terms of parasitoid oriented flights (chi-square test, χ²=65.000 df = 6, p-value < 0.001) and landings on source (chi-square test, χ²=66.022, df = 6, p-value < 0.001). The SVLM (R)-1-octen-3-ol and (R)-sulcatol elicited a statistically significant higher response in terms of oriented flights (7 and 15 times more than ethanol, respectively) and landings on source (9 and 27 times more that ethanol, respectively) by *A. ervi* in respect of ethanol (control) and respective the two (*S*)-enantiomers. The treatment with sulcatone was statistically significant compared to the control only for the oriented flights (4 times more than ethanol) of the parasitoid. The racemic mixtures of sulcatol a+b and 1-octen-3-ol d+e elicited a statistically higher response in *A. ervi* compared with the control in terms of oriented flights (14 and 6 times more than ethanol, respectively) and landings (27 and 10 more than ethanol, respectively), but not different from the respective R-enantiomers.

Following the results with single compounds and racemic mixtures we tested the 3-compounds mixture comprising only the respective active (*R*)-enantiomers and sulcatone. The mixture of (*R*)-1-octen-3-ol, (*R*)-sulcatol and sulcatone caused an average of 5-fold significant increase in parasitoid oriented flight response compared to the single compounds. Similarly, a synergistic effect of the 3-compound mixture has been observed in respect of single compounds for landings on plant, causing an average of 2.5-fold significant increase.

### 3.3 Sulcatone, sulcatol and 1-octen-3-ol impact on VOCs release

**Figure 2:**
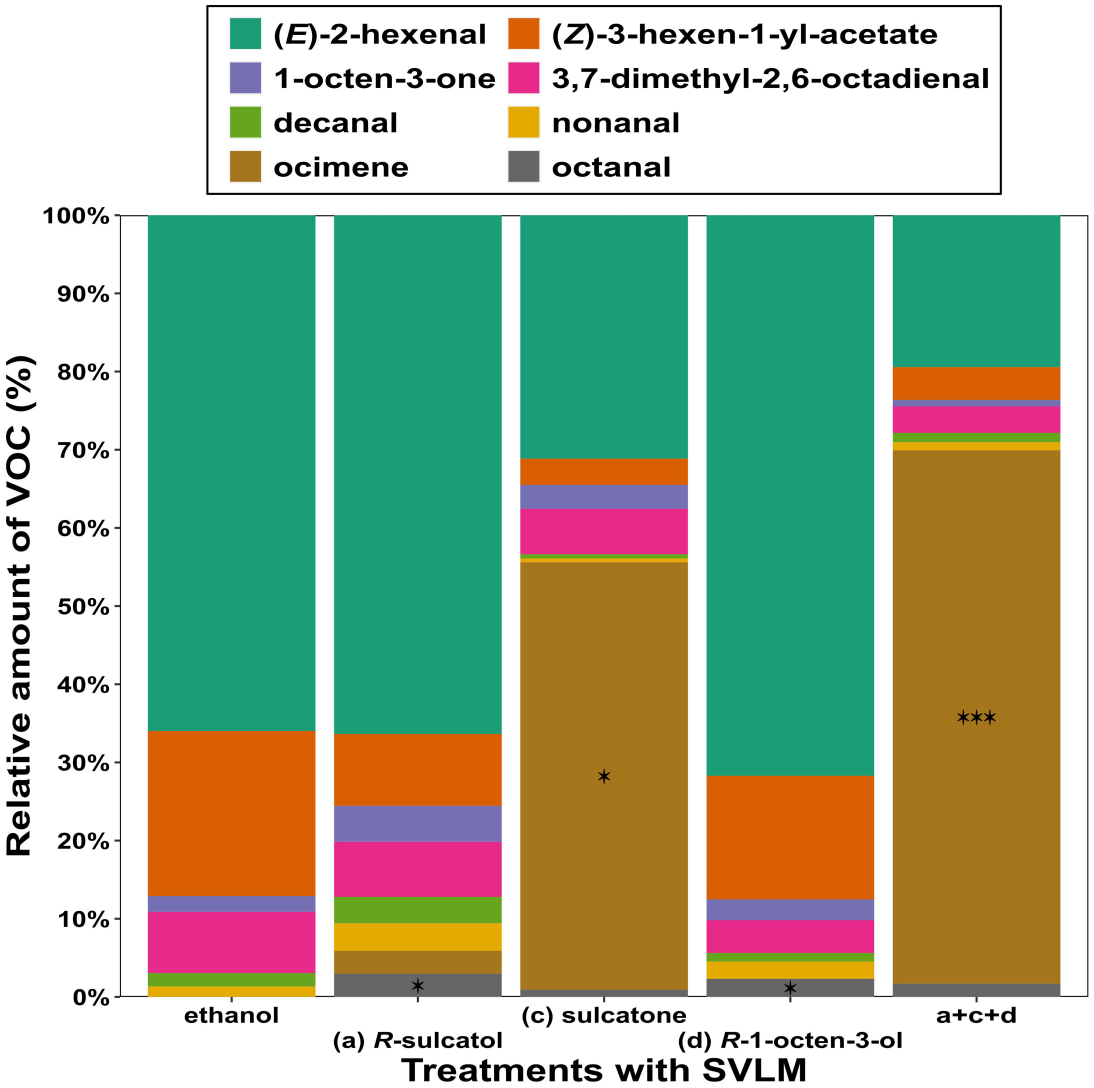
Relative amounts of VOCs emitted by *Vicia faba* plants. Different treatments with different *V. faba* SVLMs (Small Volatiles Lipophilic Molecules) and their combinations are reported on the x-axis and percentage of each VOC respect of the total VOC emission are reported on y-axis. Asterisks indicate significant difference between ethanol (control) and treatment (Dirichlet regression; *p < 0.05, ***p< 0.001).

A comparison of the proportion of the leaf VOCs emitted by *V. faba* subjected to several treatments, with the proportion of VOCs emitted by relative control plants using Dirichlet regression (**Figure 2**), showed that (*R*)-sulcatol increased the release of octanal (β = 2.145, z = 2.34, df = 33, p = 0.019), and sulcatone treatment induced a higher release of (E)-ocimene (β = 2.405, z = 2.447, df = 33, p = 0.014) compared to the control. (*R*)-1-octen-3-ol induced an increased emission of octanal (β = 2.03, z = 2.192, df = 33, p = 0.028), whereas the 3-compound mixture induce a higher release of (*E*)-ocimene (β = 3.292, z = 3.358, df = 33, p = 0.001) compared to treatments with single compounds.

**Figure 3:**
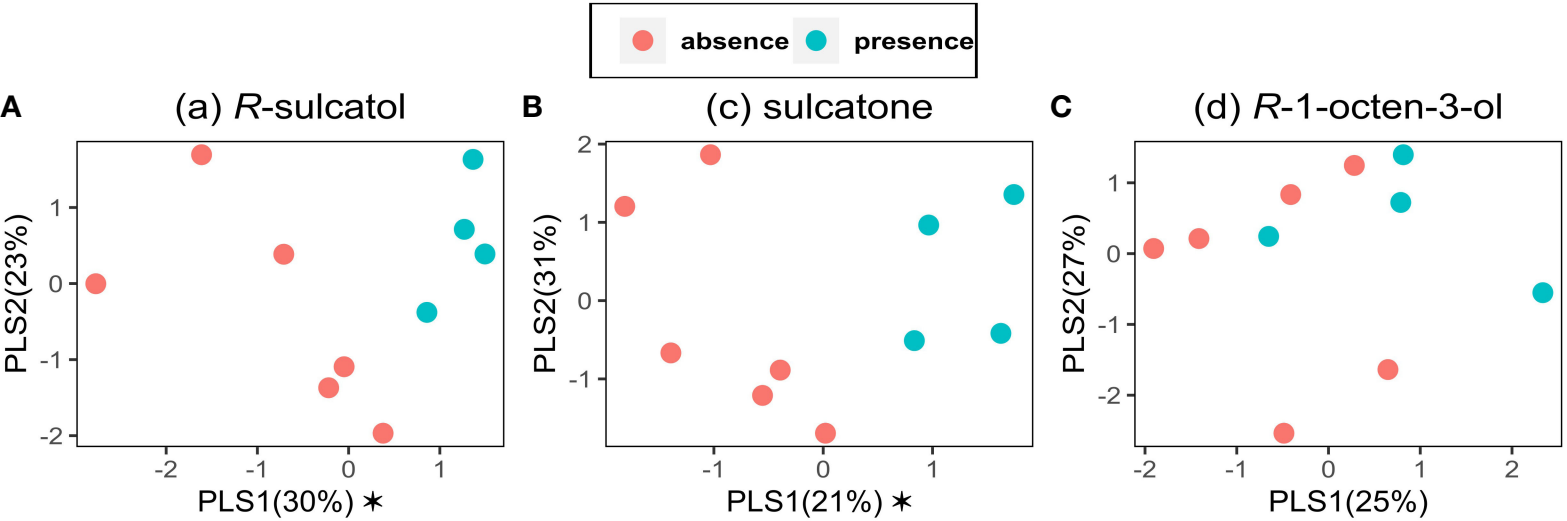
Partial Least Squares-Discriminant Analyses on VOC data. PLS-DA was performed for the VOCs emitted by V. faba plants using the presence (green points) or the absence (red points) of the treatment by the following Small Volatiles Lipophilic Molecules: **(A)** (a) *R*-sulcatol, **(B)** (c) sulcatone and **(C)** (d) *R*-1-octen-3-ol as a dependent categorical variable and VOC emission as an independent variable (n=9). Asterisk indicates a significant component (p<0.05).

Three models were built on VOCs data emitted by broad bean plants with Partial Least-Squares-Discriminant Analysis (PLS-DA), using presence and absence as dependent categorical variable (y) and VOC emission as independent variable (x). Models for each treatment with SVLM [(*R*)-sulcatol, sulcatone and (*R*)-1-octen-3-ol] are shown in **Figures 3A–C**. Using (*R*)-sulcatol as dependent variable, the model explains 53% of the total variability and gives a clear separation between treatments (**Figure 3A**), with one component being significant (R²X = 0.296, R²Y = 0.594, Q² = 0.119), while the other is shown for representational purposes (R²X = 0.228, R²Y = 0.172, Q² = 0.022). A similar separation between treatment is shown by the model using sulcatone as dependent variable. The variability explained is 52% with one significant component (R²X = 0.21, R²Y = 0.791, Q² = 0.297) and the other is shown for representational purposes (R²X = 0.306, R²Y = 0.059, Q² = -0.19). Using (*R*)-1-Octen-3-ol as dependent variable (**Figure 3C**), no significant components were found, hence this SVLM is not suitable to distinguish the VOC profile of treated plants from untreated plants.

## 4 Discussion

Aphids are oligophagous insects that feed on plants and cause damage to shoots and leaves, making them major pests across several agricultural systems, being also efficient vectors of phytopathogenic viruses (Dampc et al., 2021). As sessile organisms, plants had to evolve ways to defend themselves against herbivores (Walling, 2000), or, when in oligo-cultures, also to develop ways of transmitting signals to each other and use these signals for enhancing collective resistance (Rasmann et al., 2005; Cascone et al., 2023). From an applied perspective, the most efficient, economic and used option for protecting crops against the aphids is the repeated application of the insecticides (Schulz et al., 2021). Particularly, Imidacloprid ®, belonging to the class of insecticides called neonicotinoids, has been widely used since 1991 for the control of sucking insects such as aphids (Yuan et al., 2020; Verheggen et al., 2022). Due to adverse effects on non-target organisms, particularly on pollinators (Lu et al., 2020), the European Commission banned the outdoor use of neonicotinoids in 2018, limiting their use to permanent greenhouses (European Food Safety Authority, 2018). Therefore, an alternative to synthetic systemic formulations to control efficiently aphid pests is needed to hamper the environmental risks.

Our study tries to fill this gap, by testing alternative means of insect control. Accordingly, we took advantage of the fact that previous research has shown that different chemical elicitors can boost the innate immunity of plants, which also include the attraction of natural enemies of the herbivores near the site of wounding. Recently, we have discovered the role of a hydrophilic molecule (l-DOPA) as an anti-herbivore signal released in the aqueous phase by aphid infested broad bean plants and maybe translocated as an internal signal. Here, we have shown that three SVLMs can be similarly translocated within the rhizosphere and in the air phase contributing to plant defence (**Figures 1**, **2**), as already reported for other Volatile Organic Compounds (*e.g.* Caryophyllene) (Rasmann et al., 2005). Specifically, we hoped to find novel low molecular weight elicitors that would trigger defence responses (i.e., VOCs production from leaves) on yet-to-be attacked plants. With this aim, we started to characterize root signals released upon aphid infestation that could be exchanged belowground and alert neighbouring un-infested conspecifics to activate a defence response before the occurrence of aphid attack (Coppola et al., 2017; Takabayashi and Shiojiri, 2019).

By sampling root exudates of aphid-damaged plants, we identified three SVLMs released by *A. pisum* infested plants (**Table 1**) that are able to trigger the emission of VOCs (**Figure 2**) involved in the attraction of the aphid parasitoid *A. ervi* to uninfested plants (**Figure 1**). We have recorded the highest attraction of *A. ervi* when broad bean plants were treated with a mixture of sulcatone, sulcatol and 1-octen-3-ol. These three SVLMs are released upon aphid infestation in different plant–aphid complexes including *V. faba*. (Quiroz et al., 1997; Liu et al., 2009; Markovic et al., 2014; Dong et al., 2015; Takemoto and Takabayashi, 2015; Lin et al., 2017; Ismail et al., 2021; Higashida et al., 2022). Three results of the behavioural bioassay can be considered important: the indication of the active enantiomers of two identified SVLM (sulcatol, octen-ol); each SLVM elicited a significant higher response in terms of attraction of the natural enemy in respect of control (and non-active enantiomers); a synergistic effect was recorded when adding all 3 SVLMs.

To our knowledge, and with only two exceptions (Liu et al., 2009; Takemoto and Takabayashi, 2015), the three compounds tested in this study have been exclusively isolated from aboveground parts of the plants. All previous characterizations referred to collection that included the soil (Quiroz et al., 1997; Markovic et al., 2014; Dong et al., 2015; Lin et al., 2017; Ismail et al., 2021; Higashida et al., 2022). Hence, so far, it was not yet clearly demonstrated that sulcatone, sulcatol and 1-octen-3-ol can be released by the roots of broad bean plants, particularly in response to aphid attack, and also that they can elicit a defensive response acting belowground modifying the blend of the VOC emitted (**Figure 2**).

These three molecules were able to affect both individual VOCs and the entire volatile profile of conditioned plants. Specifically, the treatments with sulcatone and sulcatol resulted in different VOC blends, as highlighted by the PLS-DA (**Figure 3**) and supported by the behavioral observations. Conversely, R-octen-3-ol did not induce significant differences in the VOC blend (**Figure 3**), but it did in respect of the percentage of octanal (**Figure 2**).

In *V. faba*, Sulcatone (also known as 6-methyl-5-hepten-2-one) appeared in the volatile blend during the first day after *A. pisum* infestation and increased in concentration with the duration of aphid feeding (Du et al., 1998). This semiochemical is known to be biosynthesised oxidatively from the isoprenoid geraniol (Demyttenaere and De Pooter, 1996), and is probably produced through the stimulated oxidation by aphid feeding. sulcatone has been found to be a key compound in the attraction of the parasitoid *A. ervi* to plants infested by *A. pisum* (Du et al., 1998; Powell et al., 1998) and *Aphidius avenae* (Liu et al., 2001). Hence, we can here hypothesize that this compound can be actually released by both above- and belowground plant tissues. Hence, we here propose that SVLMs, when released belowground could be adsorbed by the roots of neighbouring plants, eliciting a defensive response. In support of this hypothesis, our results clearly highlighted the role played by sulcatone in triggering the release of a specific blend of VOCs, some of which are reported to be involved in the attraction of *A. ervi.* We refer in particular to the emission of (*E*)-ocimene, a strong attractant for *A. ervi* (Sasso et al., 2007; Cascone et al., 2015; Takemoto and Takabayashi, 2015), whose release is strictly correlated with the treatment with sulcatone as shown in **Figure 2**.

The presence of sulcatone in the volatile emissions of aphid-infested plants seems to be context-dependent, and varies depending on the herbivore identity. For example, the release of sulcatone was not induced by *Macrosiphum euphorbiae* feeding on tomato, although aphid-infested tomato plants were more attractive towards *A. ervi* than un-infested ones (Sasso et al., 2007). In field experiments on wheat, it was found that the rate of parasitism of the aphid *Sitobion avenae* by *Aphidius avenae* was significantly higher on plants that were sprayed with a mixture of Sulcatol and Sulcatone, compared to parasitism on plants that were sprayed with hexane (control) or not sprayed at all (Liu et al., 2009). These studies are in line with our results, where *V. faba* plants treated with Sulcatone were more attractive towards *A. ervi.* In the same way that cis-jasmone has been found to increase the attractiveness of broad bean plants towards the parasitoid *A. ervi* and to activate defence genes in broad bean plant (Birkett et al., 2000), we believe that sulcatone plays a crucial role in mediating parasitoid attraction, hence as an elicitor of parasitoid attractiveness.

Liu and collaborators (2009) showed that the parasitism level of *A. avenae* increased when the wheat plants were treated with sulcatone. Our behavioural bioassays were run 24 hours after the treatments with the SVLMs identified. Hence, the attraction of the *A. ervi* could have been influenced by the release of this compounds from the hydroponic solution. In fact, it is reasonable to exclude a direct effect of tested SVLMs on the behaviour of parasitoid *A. ervi* because we did not find it in the volatile releases of the treated plants.

Since both 1-octen-3-ol and sulcatol have asymmetrically substituted carbon atoms at positions 3 and 2 respectively, we have tested for each of both enantiomers. In fact, the link between the absolute arrangement and biological activity can vary. As an example, neither the (*R*) or (*S*) version of Sulcatol, which is a pheromone used by the ambrosia beetle *Gnathotrichus sulcatus*, has any biological activity on its own. However, when both enantiomers are combined, they produce an active mixture (Borden et al., 1976).

The third SVLM identified in the hydroponic solution of aphid-infested plants was 1-octen-3-ol, traditionally known for its mushroom-like fragrance (Wang and Zhao, 2023). This compound has been identified in the headspace of various leguminous plants (Takabayashi et al., 1991; Bendera et al., 2015; Sobhy et al., 2018). Recently, it has been identified as a key volatile compound in *V. faba* flours (Frati et al., 2017; Lu et al., 2019; Akkad et al., 2022; Duan et al., 2022). These findings are consistent with our results demonstrating that 1-octen-3-ol, a compound produced by the roots of *V. faba*, is responsible for the induction of indirect defence mechanisms against aphids. In tobacco and *Arabidopsis*, the exposure to synthetic 1-octen-3-ol has been shown to activate defence responses against *Pseudomonas syringae* and *Botrytis cinerea*, respectively (Kishimoto et al., 2007; Song et al., 2022). Theory suggests that phytopathogens and sap feeders tend to elicit similar defence responses in plants (Walling, 2000). Our findings on the role of 1-octen-3-ol reinforces this theory and reinforces the idea that this compound prompts multiple indirect defence responses (Tabata et al., 2011; Li et al., 2014; Fuchs and Krauss, 2019).

(*R*)-1-octen-3-ol is synthesized from fatty acids in cellular membranes. Since organisms need to break down intact cellular structures to produce such VOCs, there is likely a selective pressure on plants to recognize these volatiles as “danger signals” or damage-associated molecular patterns (Heil and Land, 2014; Duran-Flores and Heil, 2016) that could support the role of (*R*)-1-octen-3-ol as defence elicitor. We observed a significant behavioural response in *A. ervi* only towards plants treated with the *R*-enantiomer of both 1-Octen-3-ol and sulcatol (**Figure 1**). For these reasons, we have collected the VOCs from *V. faba* plants treated with *R*-enantiomers (**Figure 2** a+b) and in combination with the other SVLM (Figure a+c+d). For both plants treated with (*R*)-sulcatol and (*R*)-1-octen-3-ol, we have recorded a significant emission of Octanal compared to control plants, that can explain their attractiveness for A. ervi. Octanal is a specific VOC emitted by broad bean plants infested by *A. pisum* aphids and is highly attractive to *A. ervi* (Takemoto and Takabayashi, 2015).

## 5 Conclusion

Further bioassays in soil and open field are required to confirm the specificity of the defensive activation by SVLMs and then propose them as a sustainable alternative to synthetic insecticides. Moreover, it is crucial before proposing them, to study their persistence in the soil, which can be affected by abiotic (*e.g.* moisture and pH) and biotic factors (*e.g.* microbial degradation) (Som et al., 2017). Nonetheless, adding these compounds to the list of plant elicitors of direct and indirect defences will broaden the solution panel for reducing the pesticide-dependency of modern agriculture. Even if agronomic studies are needed to evaluate the putative fitness costs of the plants due to the application of these SVLMs, the application of these semiochemicals could become in the near future what has been the use of sexual pheromones for mating disruption from the late 1990s onward.

## Data availability statement

The raw data supporting the conclusions of this article will be made available by the authors, without undue reservation.

## Author contributions

EG, JP and MB conceptualization. EG, JP, MB and PC designed the research. PC performed bioassays and analysed data. JV performed VOC analysis. SR analysed data. EG, JP, SR and MB wrote the paper. All authors contributed to the article and approved the submitted version.

## References

[B1] Akkad R. Kharraz E. Han J. House J. D. Curtis J. M. (2022). The effect of short-term storage temperature on the key headspace volatile compounds observed in Canadian faba bean flour. Food Sci. Technol. Int. 28, 135–143. doi: 10.1177/1082013221998843 33653147 PMC8892053

[B2] Badri D. V. Vivanco J. M. (2009). Regulation and function of root exudates. Plant Cell Environ. 32, 666–681. doi: 10.1111/j.1365-3040.2009.01926.x 19143988

[B3] Baetz U. Martinoia E. (2014). Root exudates: the hidden part of plant defense. Trends Plant Sci. 19, 90–98. doi: 10.1016/j.tplants.2013.11.006 24332225

[B4] Bais H. P. Vepachedu R. Gilroy S. Callaway R. M. Vivanco J. M. (2003). Allelopathy and exotic plant invasion: from molecules and genes to species interactions. Science 301, 1377–1380. doi: 10.1126/science.1083245 12958360

[B5] Bais H. P. Weir T. L. Perry L. G. Gilroy S. Vivanco J. M. (2006). The role of root exudates in rhizosphere interactions with plants and other organisms. Annu. Rev. Plant Biol. 57, 233–266. doi: 10.1146/annurev.arplant.57.032905.105159 16669762

[B6] Bendera M. Ekesi S. Ndung’u M. Srinivasan R. Torto B. (2015). A major host plant volatile, 1-octen-3-ol, contributes to mating in the legume pod borer, *Maruca vitrata* (Fabricius) (Lepidoptera: crambidae). Sci. Nat. 102, 47. doi: 10.1007/s00114-015-1297-0 26280704

[B7] Biedrzycki M. L. Jilany T. A. Dudley S. A. Bais H. P. (2010). Root exudates mediate kin recognition in plants. Communicative Integr. Biol. 3, 28–35. doi: 10.4161/cib.3.1.10118 PMC2881236 20539778

[B8] Birkett M. A. Campbell C. A. Chamberlain K. Guerrieri E. Hick A. J. Martin J. L.. (2000). New roles for cis-jasmone as an insect semiochemical and in plant defense. Proc. Natl. Acad. Sci. 97, 9329–9334. doi: 10.1073/pnas.160241697 10900270 PMC16867

[B9] Birkett M. A. Pickett J. A. (2014). Prospects of genetic engineering for robust insect resistance. Curr. Opin. Plant Biol. 19, 59–67. doi: 10.1016/j.pbi.2014.03.009 24747775

[B10] Borden J. H. Chong L. McLean J. A. Slessor K. N. Mori K. (1976). *Gnathotrichus sulcatus*: synergistic response to enantiomers of the aggregation pheromone sulcatol. Science 192, 894–896. doi: 10.1126/science.1273573 1273573

[B11] Bruin J. Dicke M. (2001). Chemical information transfer between wounded and unwounded plants: backing up the future. Biochem. Systematics Ecol. 29, 1103–1113. doi: 10.1016/S0305-1978(01)00053-9

[B12] Canarini A. Kaiser C. Merchant A. Richter A. Wanek W. (2019). Root exudation of primary metabolites: mechanisms and their roles in plant responses to environmental stimuli. Front. Plant Sci. 10, 157. doi: 10.3389/fpls.2019.00157 30881364 PMC6407669

[B13] Cascone P. Iodice L. Maffei M. E. Bossi S. ichiro Arimura G. Guerrieri E. (2015). Tobacco overexpressing β-ocimene induces direct and indirect responses against aphids in receiver tomato plants. J. Plant Physiol. 173, 28–32. doi: 10.1016/j.jplph.2014.08.011 25462075

[B14] Cascone P. Vuts J. Birkett M. A. Dewhirst S. Rasmann S. Pickett J. A.. (2023). L-DOPA functions as a plant pheromone for belowground anti-herbivory communication. Ecol. Lett. 26, 460–469. doi: 10.1111/ele.14164 36708055

[B15] Chai Y. N. Schachtman D. P. (2022). Root exudates impact plant performance under abiotic stress. Trends Plant Sci. 27, 80–91. doi: 10.1016/j.tplants.2021.08.003 34481715

[B16] Chamberlain K. Guerrieri E. Pennacchio F. Pettersson J. Pickett J. A. Poppy G. M.. (2001). Can aphid-induced plant signals be transmitted aerially and through the rhizosphere? Biochem. Systematics Ecol. 29, 1063–1074. doi: 10.1016/S0305-1978(01)00050-3

[B17] Coppola M. Cascone P. Madonna V. Di Lelio I. Esposito F. Avitabile C.. (2017). Plant-to-plant communication triggered by systemin primes anti-herbivore resistance in tomato. Sci. Rep. 7, 15522. doi: 10.1038/s41598-017-15481-8 29138416 PMC5686165

[B18] Dampc J. Mołoń M. Durak T. Durak R. (2021). Changes in aphid–plant interactions under increased temperature. Biology 10, 480. doi: 10.3390/biology10060480 34071458 PMC8227038

[B19] de Mendiburu F. (2021). Agricolae: statistical procedures for agricultural research. r package version 1.3-5. 155. Available at: https://CRAN.R-project.org/package=agricolae.

[B20] Demyttenaere J. C. R. De Pooter H. L. (1996). Biotransformation of geraniol and nerol by spores of *Penicillium italicum* . Phytochemistry 41, 1079–1082. doi: 10.1016/0031-9422(95)00797-0

[B21] Dong L. Hou Y. Li F. Piao Y. Zhang X. Zhang X.. (2015). Characterization of volatile aroma compounds in different brewing barley cultivars. J. Sci. Food Agric. 95, 915–921. doi: 10.1002/jsfa.6759 24862930

[B22] Douma J. C. Weedon J. T. (2019). Analysing continuous proportions in ecology and evolution: a practical introduction to beta and dirichlet regression. Methods Ecol. Evol. 10, 1412–1430. doi: 10.1111/2041-210X.13234

[B23] Du Y. J. Poppy G. M. Powell W. Pickett J. A. Wadhams L. J. Woodcock C. M. (1998). Identification of semiochemicals released during aphid feeding that attract parasitoid *Aphidius ervi* . J. Chem. Ecol. 24, 1355–1368. doi: 10.1023/A:1021278816970

[B24] Duan S. Kwon S.-J. Gil C. S. Eom S. H. (2022). Improving the antioxidant activity and flavor of faba (*Vicia faba* l.) leaves by domestic cooking methods. Antioxidants 11, 931. doi: 10.3390/antiox11050931 35624795 PMC9137704

[B25] Duran-Flores D. Heil M. (2016). Sources of specificity in plant damaged-self recognition. Curr. Opin. Plant Biol. 32, 77–87. doi: 10.1016/j.pbi.2016.06.019 27421107

[B26] Ehlers B. K. Berg M. P. Staudt M. Holmstrup M. Glasius M. Ellers J.. (2020). Plant secondary compounds in soil and their role in belowground species interactions. Trends Ecol. Evol. 35, 716–730. doi: 10.1016/j.tree.2020.04.001 32414604

[B27] Erb M. Veyrat N. Robert C. A. Xu H. Frey M. Ton J.. (2015). Indole is an essential herbivore-induced volatile priming signal in maize. Nat. Commun. 6, 1–10. doi: 10.1038/ncomms7273 PMC4339915 25683900

[B28] European Food Safety Authority (2018) Neonicotinoids: risks to bees confirmed. Available at: https://www.efsa.europa.eu/en/press/news/180228 (Accessed January 17, 2023).

[B29] Frati F. Cusumano A. Conti E. Colazza S. Peri E. Guarino S.. (2017). Foraging behaviour of an egg parasitoid exploiting plant volatiles induced by pentatomids: the role of adaxial and abaxial leaf surfaces. PeerJ 5, e3326. doi: 10.7717/peerj.3326 28533974 PMC5437855

[B30] Fuchs B. Krauss J. (2019). Can epichloë endophytes enhance direct and indirect plant defence? Fungal Ecol. 38, 98–103. doi: 10.1016/j.funeco.2018.07.002

[B31] Fukushima J. Kuramitsu K. Takabayashi J. Kainoh Y. (2021). Delayed response after learning associated with oviposition experience in the larval parasitoid, cotesia kariyai (Hymenoptera: braconidae). J. Insect Behav. 34, 264–270. doi: 10.1007/s10905-021-09786-w

[B32] Gfeller V. Huber M. Förster C. Huang W. Köllner T. G. Erb M. (2019). Root volatiles in plant–plant interactions I: high root sesquiterpene release is associated with increased germination and growth of plant neighbours. Plant Cell Environ. 42, 1950–1963. doi: 10.1111/pce.13532 30737807 PMC6850102

[B33] Guerrieri E. Pennacchio F. Tremblay E. (1993). Flight behaviour of the aphid parasitoid *Aphidius ervi* (Hymenoptera: braconidae) in response to plant and host volatiles. Eur. J. Entomology 90, 415–421.

[B34] Guerrieri E. Poppy G. M. Powell W. Rao R. Pennacchio F. (2002). Plant-to- plant communication mediating in-flight orientation of *Aphidius ervi* . J. Chem. Ecol. 28, 1703–1715. doi: 10.1023/A:1020553531658 12449500

[B35] Guerrieri E. Poppy G. M. Powell W. Tremblay E. Pennacchio F. (1999). Induction and systemic release of herbivore-induced plant volatiles mediating in-flight orientation of aphidius ervi. J. Chem. Ecol. 25, 1247–1261. doi: 10.1023/A:1020914506782

[B36] Haichar F. e. Z. Santaella C. Heulin T. Achouak W. (2014). Root exudates mediated interactions belowground. Soil Biol. Biochem. 77, 69–80. doi: 10.1016/j.soilbio.2014.06.017

[B37] Heil M. Land W. G. (2014). Danger signals – damaged-self recognition across the tree of life. Front. Plant Sci. 5. doi: 10.3389/fpls.2014.00578 PMC4215617 25400647

[B38] Higashida K. Yano E. Takabayashi J. Ozawa R. Yoneya K. (2022). Volatiles from eggplants infested by *Aphis gossypii* induce oviposition behavior in the aphidophagous gall midge *Aphidoletes aphidimyza* . Arthropod-Plant Interact. 16, 45–52. doi: 10.1007/s11829-021-09882-w

[B39] Hu L. Robert C. A. Cadot S. Zhang X. I. Ye M. Li B.. (2018). Root exudate metabolites drive plant-soil feedbacks on growth and defense by shaping the rhizosphere microbiota. Nat. Commun. 9, 1–13. doi: 10.1038/s41467-018-05122-7 30013066 PMC6048113

[B40] Ismail M. Zanolli P. Muratori F. Hance T. (2021). Aphids facing their parasitoids: a first look at how chemical signals may make higher densities of the pea aphid *Acyrthosiphon pisum* less attractive to the parasitoid *Aphidius ervi* . Insects 12, 878. doi: 10.3390/insects12100878 34680647 PMC8538517

[B41] Johnson S. N. Rasmann S. (2015). Root-feeding insects and their interactions with organisms in the rhizosphere. Annu. Rev. Entomology 60, 517–535. doi: 10.1146/annurev-ento-010814-020608 25564744

[B42] Karban R. Yang L. H. Edwards K. F. (2014). Volatile communication between plants that affects herbivory: a meta-analysis. Ecol. Lett. 17, 44–52. doi: 10.1111/ele.12205 24165497

[B43] Kishimoto K. Matsui K. Ozawa R. Takabayashi J. (2007). Volatile 1-octen-3-ol induces a defensive response in *Arabidopsis thaliana* . J. Gen. Plant Pathol. 73, 35–37. doi: 10.1007/s10327-006-0314-8

[B44] Li T. Blande J. D. Gundel P. E. Helander M. Saikkonen K. (2014). Epichloë endophytes alter inducible indirect defences in host grasses. PloS One 9, e101331. doi: 10.1371/journal.pone.0101331 24978701 PMC4076332

[B45] Li B. Li Y.-Y. Wu H.-M. Zhang F.-F. Li C.-J. Li X.-X.. (2016). Root exudates drive interspecific facilitation by enhancing nodulation and N2 fixation. Proc. Natl. Acad. Sci. 113, 6496–6501. doi: 10.1073/pnas.1523580113 27217575 PMC4988560

[B46] Li L. Liu Y.-X. Li X.-F. (2021). “Intercropping to maximize root–root interactions in agricultural plants,” in The root systems in sustainable agricultural intensification (Chichester, West Sussex, UK: John Wiley & Sons, Ltd), 309–328. doi: 10.1002/9781119525417.ch12

[B47] Lin Y. Qasim M. Hussain M. Akutse K. S. Avery P. B. Dash C. K.. (2017). The herbivore-induced plant volatiles methyl salicylate and menthol positively affect growth and pathogenicity of entomopathogenic fungi. Sci. Rep. 7, 40494. doi: 10.1038/srep40494 28079180 PMC5227919

[B48] Liu Y. Hu C. Ni H. Sun J. (2001). Effects of volatiles from different trophic level on foraging behavior of *Aphidius avenae* . Ying Yong Sheng Tai Xue Bao 12, 581–584. 11758388

[B49] Liu Y. C. Qin X. M. Xiao J. X. Tang L. Wei C. Z. Wei J. J.. (2017). Intercropping influences component and content change of flavonoids in root exudates and nodulation of faba bean. J. Plant Interact. 12, 187–192. doi: 10.1080/17429145.2017.1308569

[B50] Liu Y. Wang W.-L. Guo G.-X. Ji X.-L. (2009). Volatile emission in wheat and parasitism by *Aphidius avenae* after exogenous application of salivary enzymes of *Sitobion avenae* . Entomologia Experimentalis Applicata 130, 215–221. doi: 10.1111/j.1570-7458.2008.00822.x

[B51] Lu Y. Chi Y. Lv Y. Yang G. He Q. (2019). Evolution of the volatile flavor compounds of Chinese horse bean-chili-paste. LWT 102, 131–135. doi: 10.1016/j.lwt.2018.12.035

[B52] Lu C. Hung Y.-T. Cheng Q. (2020). A review of sub-lethal neonicotinoid insecticides exposure and effects on pollinators. Curr. pollut. Rep. 6, 137–151. doi: 10.1007/s40726-020-00142-8

[B53] Maier M. (2014). DirichletReg: dirichlet regression for compositional data in r. (Vienna: Research Report Series). Available at: https://research.wu.ac.at/en/publications/dirichletreg-dirichlet-regression-for-compositional-data-in-r-3.

[B54] Markovic D. Glinwood R. Olsson U. Ninkovic V. (2014). Plant response to touch affects the behaviour of aphids and ladybirds. Arthropod-Plant Interact. 8, 171–181. doi: 10.1007/s11829-014-9303-6

[B55] Pickett J. A. Allemann R. K. Birkett M. A. (2013). The semiochemistry of aphids. Nat. Prod. Rep. 30, 1277–1283. doi: 10.1039/C3NP70036D 24156096

[B56] Powell W. Pennacchio F. Poppy G. M. Tremblay E. (1998). Strategies involved in the location of hosts by the parasitoid *Aphidius ervi* haliday (Hymenoptera: braconidae: aphidiinae). Biol. Control 11, 104–112. doi: 10.1006/bcon.1997.0584

[B57] Quiroz A. Pettersson J. Pickett J. A. Wadhams L. J. Niemeyer H. M. (1997). Semiochemicals mediating spacing behavior of bird cherry-oat aphid, *Rhopalosiphum padi* feeding on cereals. J. Chem. Ecol. 23, 2599–2607. doi: 10.1023/B:JOEC.0000006669.34845.0d

[B58] Rasmann S. Hiltpold I. (2022). Root exudation of specialized molecules for plant-environment interaction. Chimia 76, 922–922. doi: 10.2533/chimia.2022.922 38069787

[B59] Rasmann S. Köllner T. G. Degenhardt J. Hiltpold I. Toepfer S. Kuhlmann U.. (2005). Recruitment of entomopathogenic nematodes by insect-damaged maize roots. Nature 434, 732–737. doi: 10.1038/nature03451 15815622

[B60] Rasmann S. Turlings T. C. (2016). Root signals that mediate mutualistic interactions in the rhizosphere. Curr. Opin. Plant Biol. 32, 62–68. doi: 10.1016/j.pbi.2016.06.017 27393937

[B61] R Core Team (2022) R: a language and environment for statistical computing. Available at: https://www.r-project.org.

[B62] Robert C. A. Erb M. Duployer M. Zwahlen C. Doyen G. R. Turlings T. C. (2012). Herbivore-induced plant volatiles mediate host selection by a root herbivore. New Phytol. 194, 1061–1069. doi: 10.1111/j.1469-8137.2012.04127.x 22486361

[B63] Rovira A. D. (1969). Plant root exudates. Bot. Rev. 35, 35–57. doi: 10.1007/BF02859887

[B64] Sasso R. Iodice L. Cristina Digilio M. Carretta A. Ariati L. Guerrieri E. (2007). Host-locating response by the aphid parasitoid *Aphidius ervi* to tomato plant volatiles. J. Plant Interact. 2, 175–183. doi: 10.1080/17429140701591951

[B65] Schulz R. Bub S. Petschick L. L. Stehle S. Wolfram J. (2021). Applied pesticide toxicity shifts toward plants and invertebrates, even in GM crops. Science 372, 81–84. doi: 10.1126/science.abe1148 33795455

[B66] Sharifi R. Ryu C.-M. (2021). Social networking in crop plants: wired and wireless cross-plant communications. Plant Cell Environ. 44, 1095–1110. doi: 10.1111/pce.13966 33274469 PMC8049059

[B67] Sobhy I. S. Bruce T. J. Turlings T. C. (2018). Priming of cowpea volatile emissions with defense inducers enhances the plant’s attractiveness to parasitoids when attacked by caterpillars. Pest Manage. Sci. 74, 966–977. doi: 10.1002/ps.4796 29155489

[B68] Som S. Willett D. S. Alborn H. T. (2017). Dynamics of belowground volatile diffusion and degradation. Rhizosphere 4, 70–74. doi: 10.1016/j.rhisph.2017.07.004

[B69] Song G. C. Jeon J.-S. Choi H. K. Sim H.-J. Kim S.-G. Ryu C.-M. (2022). Bacterial type III effector–induced plant C8 volatiles elicit antibacterial immunity in heterospecific neighbouring plants via airborne signalling. Plant Cell Environ. 45, 236–247. doi: 10.1111/pce.14209 34708407 PMC9298316

[B70] Tabata J. Moraes C. M. D. Mescher M. C. (2011). Olfactory cues from plants infected by powdery mildew guide foraging by a mycophagous ladybird beetle. PloS One 6, e23799. doi: 10.1371/journal.pone.0023799 21876772 PMC3158101

[B71] Takabayashi J. Dicke M. Posthumus M. A. (1991). Variation in composition of predator-attracting allelochemicals emitted by herbivore-infested plants: relative influence of plant and herbivore. Chemoecology 2, 1–6. doi: 10.1007/BF01240659

[B72] Takabayashi J. Shiojiri K. (2019). Multifunctionality of herbivory-induced plant volatiles in chemical communication in tritrophic interactions. Curr. Opin. Insect Sci. 32, 110–117. doi: 10.1016/j.cois.2019.01.003 31113622

[B73] Takemoto H. Takabayashi J. (2015). Parasitic wasps *Aphidius ervi* are more attracted to a blend of host-induced plant volatiles than to the independent compounds. J. Chem. Ecol. 41, 801–807. doi: 10.1007/s10886-015-0615-5 26302986

[B74] Thévenot E. A. Roux A. Xu Y. Ezan E. Junot C. (2015). Analysis of the human adult urinary metabolome variations with age, body mass index, and gender by implementing a comprehensive workflow for univariate and OPLS statistical analyse. J. Proteome Res. 14, 3322–3335. doi: 10.1021/acs.jproteome.5b00354 26088811

[B75] UE (2009). Directive 2009/128/EC of the European parliament and of the council of 21 October 2009 establishing a framework for community action to achieve the sustainable use of pesticides. Off J. Eur. Union L309, 71–86.

[B76] Van Emden R. H. H. F. van Emden H. F. F. Harrington R. (2007). Aphids as crop pests. Eds. van Emden H. F. Harrington R. (Wallingford: CABI). doi: 10.1086/596270

[B77] Verheggen F. Barrès B. Bonafos R. Desneux N. Escobar-Gutiérrez A. J. Gachet E.. (2022). Producing sugar beets without neonicotinoids: an evaluation of alternatives for the management of viruses-transmitting aphids. entomologia 42, 491–498. doi: 10.1127/entomologia/2022/1511

[B78] Walker T. S. Bais H. P. Grotewold E. Vivanco J. M. (2003). Root exudation and rhizosphere biology. Plant Physiol. 132, 44–51. doi: 10.1104/pp.102.019661 12746510 PMC1540314

[B79] Walling L. L. (2000). The myriad plant responses to herbivores. J. Plant Growth Regul. 19, 195–216. doi: 10.1007/s003440000026 11038228

[B80] Wang M. Zhao R. (2023). A review on nutritional advantages of edible mushrooms and its industrialization development situation in protein meat analogues. J. Future Foods 3, 1–7. doi: 10.1016/j.jfutfo.2022.09.001

[B81] Weir T. L. Perry L. G. Gilroy S. Vivanco J. M. (2010). The role of root exudates in rhizosphere interactions with plants and other organisms. Annu. Rev. Plant Biol. 57, 233–66. doi: 10.1146/annurev-plant-57-033010-200001 16669762

[B82] Weisskopf L. Ryu C.-M. Raaijmakers J. Garbeva P. (2016). Editorial: smelly fumes - volatile-mediated communication between bacteria and other organisms. Front. Microbiol. 7. doi: 10.3389/fmicb.2016.02031 PMC5168464 28066357

[B83] Yuan W. Xu B. Ran G. Chen H. Zhao P. Huang Q. (2020). Application of imidacloprid controlled-release granules to enhance the utilization rate and control wheat aphid on winter wheat. J. Integr. Agric. 19, 3045–3053. doi: 10.1016/S2095-3119(20)63240-3

