# Supplementary material for: Small volatile lipophilic molecules induced belowground by aphid attack elicit a defensive response in neighbouring un-infested plants
Source: Front Plant Sci. 2023 Jun 23;14:1154587. doi: 10.3389/fpls.2023.1154587 (PMC10326905; doi:10.3389/fpls.2023.1154587)
Supplement: Supplementary file 1 [file DataSheet_1.docx]

Supplementary Material

Small Volatile Lipophilic Molecules induced by Aphid attack elicit a Defensive Response in neighbouring un-infested belowground plants

Pasquale Cascone^1^, Jozsef Vuts^2^, Michael A. Birkett^2^, Sergio Rasmann^3^, John A. Pickett^4^, Emilio Guerrieri^1,5^*

^1^Institute for Sustainable Plant Protection, Consiglio Nazionale delle Ricerche, P.le Enrico Fermi 1, 80055 Portici, Napoli, Italy

^2^Biointeractions and Crop Protection Department, Rothamsted Research, Harpenden, Hertfordshire, AL5 2JQ, United Kingdom

^3^Institute of Biology, University of Neuchatel, Rue Emile-Argand 11, 2000 Neuchatel, Switzerland

^4^School of Chemistry, Cardiff University, Cardiff, CF10 3AT, United Kingdom

^5^Institute for Sustainable Plant Protection, Consiglio Nazionale delle Ricerche, Strada delle Cacce 73, 10135 Torino, Italy

***Correspondence:**Corresponding Author
emilio.guerrieri@ipsp.cnr.it

# Supplementary Figures

## Figure Legends

**Fig S1. GC traces of ether extracts of *V. faba* hydroponics.** Top: control plants, bottom: aphid-infested plants. Asterisks mark Sulcatone, 1-Octen-3-ol and Sulcatol.

**- Figure S1-**

**
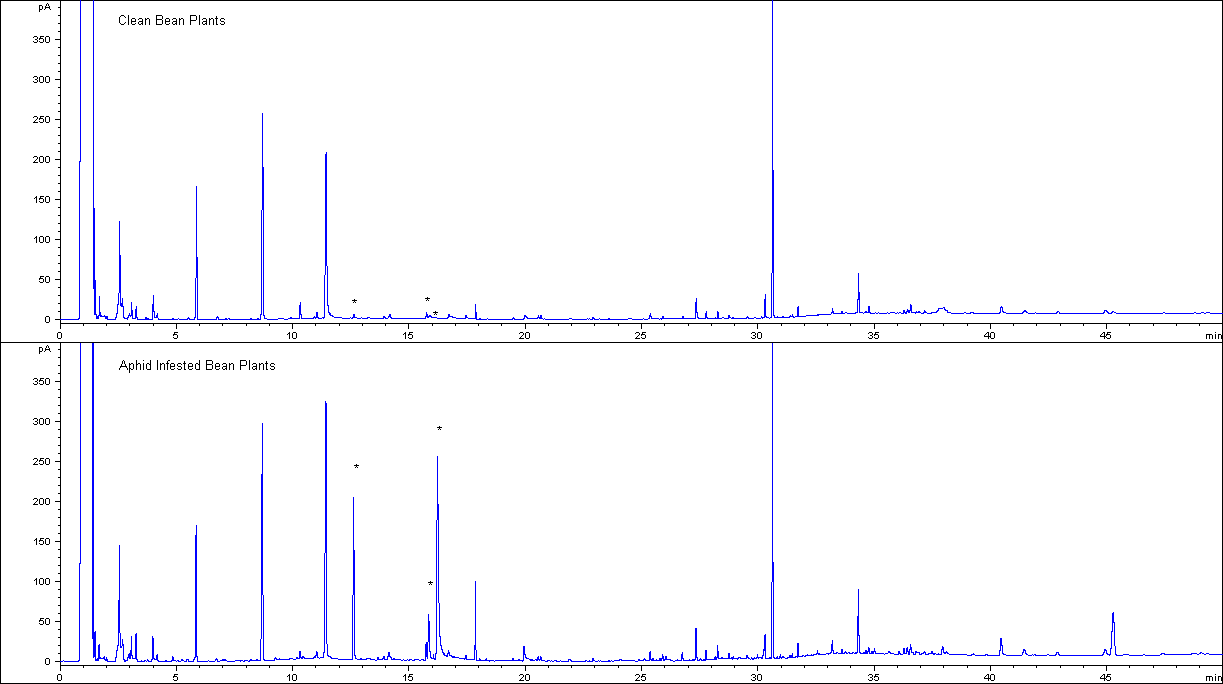
**
